# Preparation, Characterisation, and Topical Delivery of Terbinafine

**DOI:** 10.3390/pharmaceutics11100548

**Published:** 2019-10-22

**Authors:** A. S. M. Monjur Al Hossain, Bruno C. Sil, Fotis Iliopoulos, Rebecca Lever, Jonathan Hadgraft, Majella E. Lane

**Affiliations:** 1 UCL School of Pharmacy, 29-39 Brunswick Square, London WC1N 1AX, UK; fotis.iliopoulos.16@ucl.ac.uk (F.I.); rebecca.lever@ucl.ac.uk (R.L.); jonathan.hadgraft@btinternet.com (J.H.); m.lane@ucl.ac.uk (M.E.L.); 2 Department of Pharmaceutical Technology, Faculty of Pharmacy, University of Dhaka, Dhaka-1000, Bangladesh; 3 Department of Pharmaceutical Science and Pharmacology, London Metropolitan University, 166–220 Holloway Road, London N7 8DB, UK; b.dasilvasildossantos@londonmet.ac.uk

**Keywords:** TBF, fungal infections, topical delivery, in vitro, porcine skin

## Abstract

Terbinafine (TBF) is commonly used in the management of fungal infections of the skin because of its broad spectrum of activity. Currently, formulations containing the free base and salt form are available. However, there is only limited information in the literature about the physicochemical properties of this drug and its uptake by the skin. In this work, we conducted a comprehensive characterisation of TBF, and we also examined its percutaneous absorption in vitro in porcine skin. TBF-free base was synthesised from the hydrochloride salt by a simple proton displacement reaction. Both the free base and salt form were further analysed using Differential Scanning Calorimetry (DSC) and Thermogravimetric Analysis (TGA). Delivery of TBF-free base in excised porcine skin was investigated from the following solvents: Isopropyl myristate (IPM), propylene glycol monolaurate (PGML), Transcutol^®^ (TC), propylene glycol (PG), polyethylene glycol 200 (PEG 200), oleic acid (OL), ethanol (EtOH), and isopropyl alcohol (IPA). Permeation and mass balance studies confirmed that PG and TC were the most efficacious vehicles, delivering higher amounts of TBF-free base to the skin compared with a commercial gel (*p* < 0.05). These preliminary results are promising and will inform the development of more complex formulations in future work.

## 1. Introduction

Fungal skin infections are reported to be the most common skin disease, and were ranked in the top 10 most prevalent diseases worldwide in 2010 [1,2,3,4]. About 25% of the world’s population has also been reported to have fungal infections of the skin or nails of the foot [2,5]. These infections are often seen in the outer layers of the skin, the nails, and hair [3]. Unlike many other infections affecting the body, fungi may cause dermatological conditions that do not involve tissue invasion [6]. The skin between the toes is also more prone to fungal infection (athlete’s foot) and inflammation and itchiness are pervasive problems in this case [7]. The infected skin becomes red, cracked, white, and softened. Small blisters may also appear, which can burst and spread to other parts of the body [8]. Currently, there is no consensus with regard to the optimal period of treatment, drug dose, and frequency of application of topical preparations of antifungal agents [8].

Terbinafine (TBF) is a lipophilic antifungal drug that was first licensed for the management of fungal infections in the early 1990s [9]. Structurally, TBF is an allylamine with the chemical formula C_21_H_25_N, a molecular weight of 291.4 g/mol, and a LogP_(octanol/water)_ (octanol-water partition coefficient) value of 6.0 [10]. The molecule acts by inhibiting squalene epoxidase in the fungal biosynthesis of ergosterol, which is an essential component for the growth of fungal cell membranes. The blockage of squalene monooxygenase by TBF at the point of squalene epoxidation results in an accumulation of squalene, which is considered to be responsible for the antifungal activity of TBF [11].

TBF has a broad spectrum of antifungal activity against fungal infections of the skin. Primarily, TBF exerts fungicidal activity against dermatophytes, while a fungistatic activity is seen against *C. albicans* [12]. The minimum inhibitory concentration (MIC) of TBF against dermatophytes such as *Trichophyton*, *Microsporum*, and *Epidermophyton* spp. ranges between 0.001–0.05 µg/mL [13,14,15,16]. Thus, TBF is more potent than azole derivatives, for which reported MIC values range from 0.1 to >10 µg/mL [16,17]. The in vitro activity of TBF against dermatophytes, as a rule, is reported to exceed the activity of other antifungal agents [13,16,18].

Surprisingly, the literature is scarce with reference to the physicochemical properties of TBF, as well as studies on skin penetration of the drug. Currently, both the free base and hydrochloride salt are available in topical formulations, despite the former having the preferred properties for skin delivery. The objectives of the present work were to (1) characterise the relevant physical and chemical properties of TBF-free base for topical delivery, and (2) examine the delivery of TBF-free base from simple solvents compared with a commercial control. The solvents were chosen based on the range of their solubility parameter values and their favourable safety profile [19,20], and included isopropyl myristate (IPM), Lauroglycol™ 90 (propylene glycol monolaurate (PGML)), Transcutol^®^ (TC), propylene glycol (PG), polyethylene glycol 200 (PEG 200), oleic acid (OL), ethanol (EtOH), and isopropyl alcohol (IPA).

## 2. Materials and Methods

### 2.1. Materials

TBF hydrochloride was donated by GSK Ltd. (Weybridge, UK), while TBF-free base was synthesised, purified, and characterised in house. Dichloromethane, sodium carbonate, magnesium sulfate, polyethylene glycol 200 (PEG 200), HPLC grade water, isopropyl alcohol (IPA), and absolute ethanol (EtOH) were supplied by Fisher Scientific (Leicestershire, UK). HPLC grade solvents, including methanol and polyoxyethylene (20) oleyl ether (Brij^®^ O20) were obtained from Sigma-Aldrich (Dorset, UK). PG and IPM (analytical grade) were purchased from Alfa Aesar (Thermo Fisher Scientific, Lancashire, UK). TC and PGML (analytical grade) were gifts from Gattefossé (St. Priest, France). Trifluoroacetic acid was supplied by Acros Organics (Fisher Scientific, Leicestershire, UK) and OL from Fluka (Sigma-Aldrich, Dorset, UK). Chloroform-D (CDCl_3_) was obtained from Cambridge Isotope Laboratories, Inc. (Andover, MA, USA). Phosphate buffered saline (PBS) tablets (Dulbecco A, pH 7.3 ± 0.2 at 25 °C) were purchased from Oxoid Limited (Cheshire, UK), and high-vacuum grease was obtained from Dow Corning (Seneffe, Belgium). Lamisil^®^ AT gel (TBF 1% *w*/*w*, GlaxoSmithKline, Brentford, UK) was purchased from a local pharmacy. Porcine ear skin was sourced from a local abattoir.

### 2.2. Methods

#### 2.2.1. Conversion of TBF Hydrochloride to TBF-Free Base

TBF hydrochloride (10 g; 0.03 mmol) was dissolved in a 70 mL biphasic mixture of HPLC water and dichloromethane (DCM) (47:53), and to this, solid sodium carbonate (1.87 g) was added. The mixture was then stirred for 1 h at 20–25 °C. After this period, the organic layer was separated, and the aqueous layer washed three times with DCM (3 × 5 mL). The organic layers were then combined and dried with anhydrous magnesium sulphate. Subsequently, the solvent was evaporated with a rotary evaporator (Heidolph, Schwabach, Germany), leaving TBF as the free base (8.2 g; 0.028 mmol) with the appearance of a thick oil at a yield of 93.8%. The oil was treated with methanol and freeze-dried (Heto Power Dry LL 1500, Thermo Electron Corporation, Warwickshire, UK) to yield TBF base as a white solid. The methodology was adapted from Korean patent publication no. KR100979903B1 with slight modifications [21]. NMR analysis was subsequently carried out with a Bruker Avance 500 MHz NMR (Bruker Corporation, Billerica, MA, USA) equipped with broadband and selective (^1^*H*) inverse probes. The analysis was conducted at a temperature of 300 K and chloroform-D (CDCl_3_) was used as the solvent.

#### 2.2.2. Differential Scanning Calorimetry (DSC) and Thermogravimetric Analysis (TGA)

The degradation temperature and melting point of TBF-free base and salt were determined using thermogravimetric analysis (TGA) and differential scanning calorimetry (DSC). The methods followed those described by Parisi et al. with slight modifications [22]. For the TGA experiment, a Discovery TGA (TA Instruments - Waters LLC, New Castle, DE, USA) system was used. Both the actives were weighed in an open tared aluminium pan (TA Instruments, New Castle, DE, USA) and then heated in the Discovery TGA furnace from 25 to 400 °C for the TBF-free base and from 25 to 500 °C for the salt, respectively. A heating ramp of 20 °C/min with a constant nitrogen flow of 25 mL/min was used throughout the experiment.

The melting point of both free base and salt forms of TBF were measured with a DSC Q2000 (TA Instruments, New Castle, DE, USA) system. The samples were placed in Tzero aluminium pans (TA Instruments, New Castle, DE, USA) which were sealed with Tzero aluminium lids (TA Instruments, New Castle, DE, USA) using a Tzero™ press (TA Instruments, New Castle, DE, USA). The sealed pans containing samples were weighed. An empty Tzero aluminium pan sealed with a Tzero aluminium lid was also weighed, which was further used as a reference. Both the sample and the reference pans were then heated inside the DSC Q2000 furnace from 0 to 150 °C for the TBF-free base compound, and from 0 to 230 °C for the TBF salt form. A heating ramp of 10 °C/min and a continuous nitrogen flow of 50 mL/min were used throughout the experiment.

#### 2.2.3. HPLC Analysis and Method Validation

An HPLC system (Agilent Technologies 1260 series, Santa Clara, CA, USA) coupled with a Shiseido MGII Capcel Pack C_18_, 5 μm, 4.6 mm × 250 mm column fitted with a universal guard column containing a C_18_ cartridge (Phenomenex, Macclesfield, UK) was used throughout the experimental analysis of TBF-free base. The mobile phase consisted of 0.1% trifluoroacetic acid (TFA) in water:methanol (30:70, *v*/*v*) with a flow rate of 1 mL/min. The ultraviolet (UV) wavelength was fixed at 225 nm. The column temperature and injection volume were fixed at 30 °C and 5 μL, respectively, for the total run time of 8 min. ChemStation^®^ for LC 3D Rev. A. 09.03 software (Agilent Technologies, Santa Clara, CA, USA) was used to acquire and analyse data. Validation parameters such as linearity, accuracy, precision, lower limit of detection (LOD), and lower limit of quantification (LOQ) were established according to the International Conference on Harmonisation (ICH) guidelines [23]. Calibration curves of TBF base were constructed from 0.5 to 50 μg/mL. Good linearity was observed within the concentration range, as the regression coefficient (R^2^) values were greater than 0.99. The HPLC methods showed an accuracy value of 101.5 ± 3.3%. In addition, the % RSD for the intraday and interday precision values were less than 0.75% and 1.4%, respectively. The limit of detection (LOD) and limit of quantification (LOQ) were 0.4 μg/mL and 1.21 μg/mL, respectively.

#### 2.2.4. Solubility Parameters, Solubility, and Stability Studies

The Molecular Modeling Pro^®^ Plus software (Version 7.0.8, Norgwyn Montgomery Software Inc., North Wales, PA, USA, 2016) was used to determine the van Krevelen and Hoftyzer’s solubility parameters of the TBF-free base compound and solvents. The solubility of TBF-free base was evaluated in solvents at 32 ± 1 °C (the surface temperature of skin). Then, 0.05 mL of each solvent was taken in a 1.5 mL Eppendorf^®^ tube in triplicate, and an excess amount of TBF-free base was added. The tubes sealed with Parafilm^®^ were then placed in a thermostatically controlled orbital shaker (Orbital Mini shaker, VWR International Limited, Leicestershire, UK) with 1000 rpm rotation for 48 h. The tubes were monitored from time to time to confirm the visible excess amount of the drug. After the 48 h period, tubes were centrifuged at 13,200 rpm for 20 min at 32 ± 1 °C in an Eppendorf 5415R centrifuge (Eppendorf, Hamburg, Germany). Finally, the concentration of the active was determined from the diluted supernatant solution using the validated HPLC method as mentioned in Section 2.2.4.

The stability of the TBF-free base in several solvent systems was measured over 120 h at 32 ± 1 °C. Known concentrations of TBF in different solvents were prepared in triplicate and placed in small glass vials. The vials were shaken for 5 min and subsequently filtered using a Millex^®^ Gp filter unit (0.22 μm, Millipore Express^®^, Carrigtwohill, Ireland). The solution was then placed in a glass vial with screw cap and sealed with Parafilm^®^. The glass vials were placed in a SUB 28 temperature-controlled water bath (Grant Instruments, Cambridge, UK) with magnetic stir beads for 120 h at 32 ± 1 °C. The samples were collected every 24 h (0, 24, 48, 72, 96, and 120 h) and diluted accordingly. Finally, the concentration of the active was determined by HPLC and expressed as a function of the concentration at 0 h.

#### 2.2.5. Dynamic Vapour Sorption Studies

Dynamic vapour sorption (DVS) studies were conducted for 24 h to examine evaporation of candidate formulations. A DVS instrument Q5000 SA (TA Instruments, New Castle, DE, USA) was used with nitrogen as the carrier gas at a flow rate of 200 mL/min. Temperature and relative humidity (RH) were maintained at 32 ± 1 °C and 50 ± 1%. Then, 10 µL of the various formulations, i.e., 1% TBF (*w*/*w*) in IPM, PGML, TC, PG, OL, and PEG 200, were applied to the sample pan and equilibrated for approximately 1 min. TA Instruments sorption analyser software was used to record the weight of the sample over 24 h. The mass changes at different time intervals (0, 0.25, 0.5, 1, 2, 4, 6, 8, 10, 12, 14, 16, 18, 20, 22, and 24 h) because of evaporation or hydration were calculated to determine amounts of formulation remaining (%). The number of replicate experiments was *n* = 3.

#### 2.2.6. Permeation and Mass Balance Studies of TBF-Free Base (1% *w*/*w*) in Porcine Skin

In vitro permeation studies of TBF-free base in Franz diffusion cells from selected single solvents were performed using full-thickness porcine ear skin. The skin was separated from the cartilage as reported previously [24]. For permeation studies, 1% (*w*/*w*) formulations of TBF-free base in the selected solvents were used as the donor solution, as well as a commercial control (Lamisil^®^ AT gel, 1% *w*/*w*). The exact diameters of the donor and receptor areas were measured before assembling the cells. The full-thickness porcine skin was cut to appropriate sizes using a manual cutter. The skin was placed between the donor and receptor compartment with the epidermis facing upwards. Freshly prepared 6% Brij^®^ O20 PBS solution was used as the receptor solution for TBF [25]. The solubility of TBF in 6% Brij^®^ O20 (*w*/*v*) was measured in order to confirm sink conditions [26]. A magnetic stir bead was inserted into the receptor compartment to ensure uniform mixing of all components with the receptor solution, and the cells were placed in a temperature-controlled water bath for 30 min to 1 h. Once the skin temperature reached 32 ± 1 °C, a 10 μL aliquot of the formulation was applied on porcine skin (approximately 1 cm^2^, but the known area was determined accurately) using an Eppendorf^®^ Multipipette Plus (Eppendorf, Hamburg, Germany). The donor compartment was unoccluded for all experiments. A volume of 200 μL of receptor solution was withdrawn from the receptor compartment at different time intervals up to 24 h, replacing the same volume with fresh temperature equilibrated receptor solution. All the samples were analysed using HPLC.

After the finite dose permeation studies, validated mass balance studies were conducted to determine the amount of TBF remaining on the skin surface and the amount delivered into the skin. The skin surface was washed five consecutive times, with 1 mL of methanol followed by a cotton bud swabbing. The samples were placed in separate Eppendorf^®^ tubes and mixed using a Vortex mixer (IKA vortex mixer genius 3, VWR International Limited, Leicestershire, UK) for 10 min at room temperature. The Franz cells were disassembled, and skin was then cut into small pieces with scissors and placed in 2 mL Eppendorf^®^ tubes with 1 mL of methanol. The tubes were placed in an orbital shaker (1000 rpm) at 32 °C for 20 h to conduct skin extraction. All the tubes containing skin and washing samples were centrifuged at 13,200 rpm, at 32 °C for 15 min. The supernatant solution was then diluted where necessary and analysed using the validated HPLC method. The reliability of the procedure was confirmed by the total active ingredient recovery (%) which should be within the range of 80 to 120% [27].

#### 2.2.7. Data Treatment and Statistical Analysis

Data treatment was performed using Microsoft^®^ Excel 2013 (Microsoft Corporation, Redmond, Washington, USA). The calculated mean refers to the arithmetic average (sum of the variables divided by the number of observations), while the standard deviation calculation was based on the *n*−1 method (*n* = small number of observations). GraphPad Prism Statistics software (version 8.1.1, San Diego, CA, USA, 2019) was used for statistical evaluation. For ≥3 groups, one-way analysis of variance (ANOVA) was used as a parametric test. Multiple comparisons between each individual group were performed by a post-hoc Tukey test. For non-parametric data, the Kruskal-Wallis test was performed. A value of *p* < 0.05 was considered a statistically significant difference.

## 3. Results and Discussions

### 3.1. Conversion of TBF Salt to the Base Form

**Figure 1:**
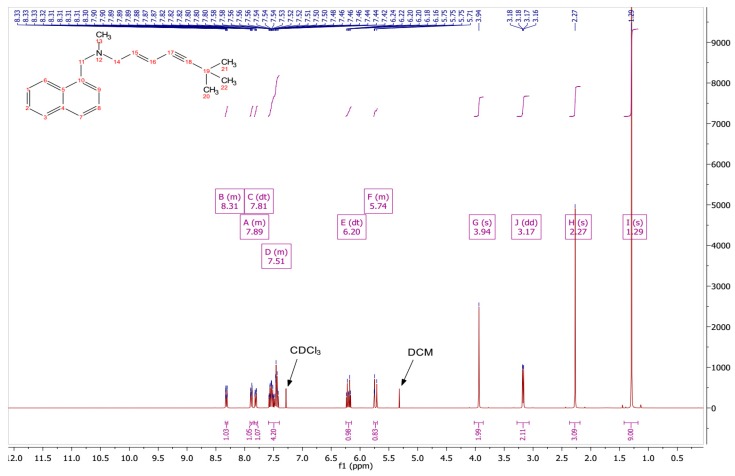
^1^*H* NMR spectrum of TBF-free base.

**Figure 2:**
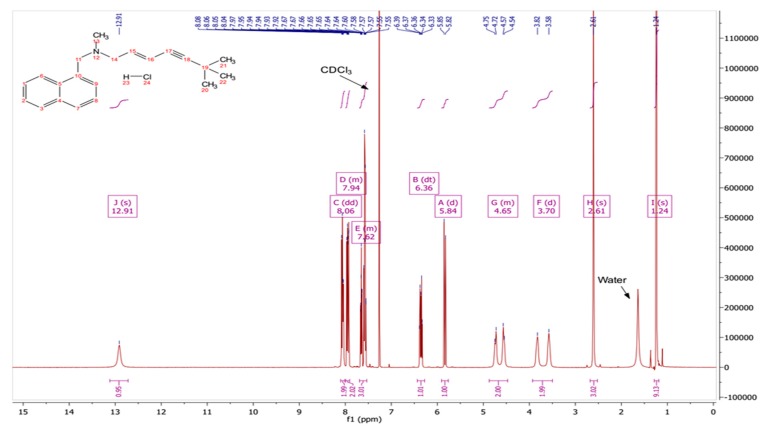
^1^*H* NMR spectrum of TBF HCl.

The conversion to the free base was confirmed by comparing experimental ^1^*H* NMR data (Figure 1) with the data reported for the ^1^*H* NMR analysis of TBF-free base reported in United States Patent Publication No.: US 6,515,181 B2 [28]. In addition, the results for the ^1^*H* NMR analysis of the starting material, TBF hydrochloride, are shown in Figure 2, with a singlet peak at 12.91 ppm for the hydrochloride proton, which is absent in the ^1^*H* NMR spectrum of the base. The spectral data are as follows:

^1^*H* NMR (500 MHz, Chloroform-d, δ in ppm) δ 8.34–8.29 (m, 1H, Ar–*H*), 7.91–7.85 (m, 2H, Ar–*H*), 7.59–7.40 (m, 4H, Ar–*H*), 6.20–6.24 (m, 1H, C*H*_2_), 5.76–5.70 (m, 1H, C*H*), 3.94 (s, 2H, N–C*H*_2_–Ar), 3.17 (dd, J = 6.6, 2H, CH–C*H*_2_–N), 2.27 (s, 3H, N–C*H*_3_), 1.29 (s, 9H, 3 × C*H*_3_).

^13^**C** NMR of TBF base (101 MHz, Chloroform-d, δ in ppm) δ 139.45 (**C**H–CH_2_), 134.94 (Ar–**C**–H), 134.01 (Ar–**C**–H), 132.58 (Ar–**C**–H), 128.53 (Ar–**C**–H), 128.03 (Ar–**C**–H), 127.37 (Ar–**C**–H), 125.93 (Ar–**C**–H), 125.70 (Ar–**C**–H), 125.23 (Ar–**C**–H), 124.78 (Ar–**C**–H), 112.91(**C**H–C≡C), 98.52 (Quaternary **C**), 77.35 (**C**≡C), 60.18 (–**C**H_2_), 59.79 (–**C**H_2_), 42.45 (N–**C**H_3_), 31.17 (3 × **C**H_3_), 28.03 (Quaternary **C**).

The data for ^13^**C** NMR analysis of both base and salt indicate no difference between the spectra, as there is no structural change after the conversion (Figures S1 and S2).

### 3.2. TGA and DSC Analysis of TBF-Free Base

**Figure 3:**
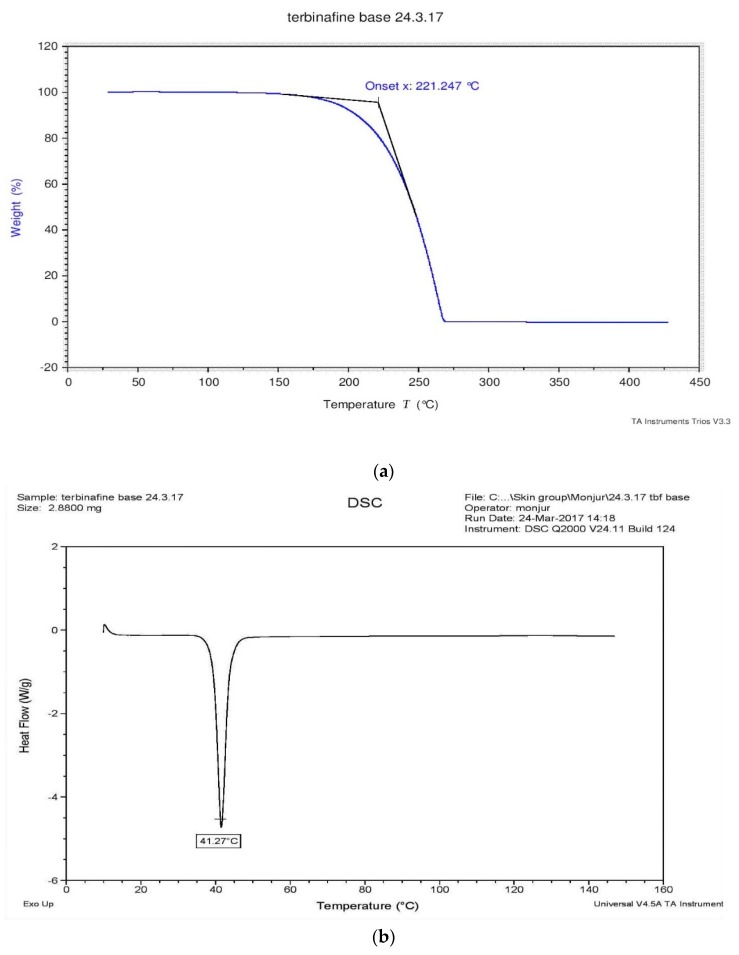
TGA (**a**) and DSC (**b**) analysis of TBF-free base.

The results for the TGA and the DSC analysis of the TBF-free base are shown in Figure 3. The TGA results indicate that no water loss occurs between 25 and 200 °C and degradation is evident between 200 and 280 °C, specifically at 221.25 °C. The DSC analysis shows one endothermic event, which indicates that the melting point of the TBF-free base is 41.27 °C. The degradation temperature and melting point of the TBF salt are 229.48 °C and 213.5 °C, respectively (Figure S3).

### 3.3. Solubility Parameters, Solubility, and Stability Studies

**Figure 4:**
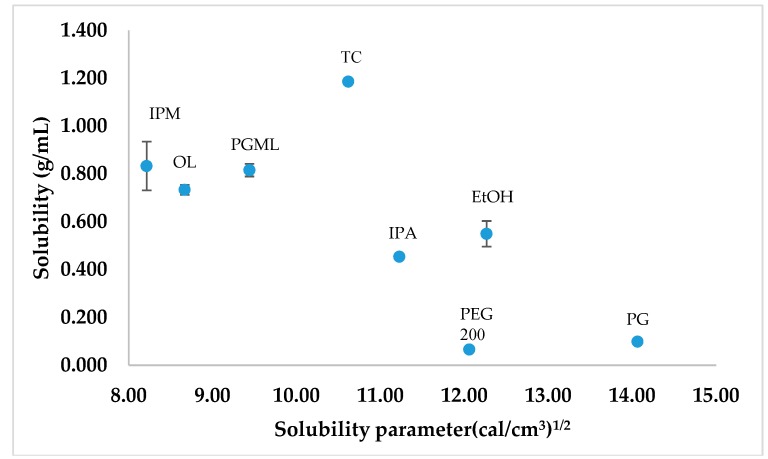
The calculated solubility parameters of different solvents plotted against the corresponding saturation solubility of TBF-free base at 32 ± 1 °C (*n* = 3, mean ± SD).

The van Krevelen and Hoftyzer solubility parameters of the various solvents were plotted against their corresponding TBF-free base solubility (Figure 4). The solubility of a solute in the respective solvent has been suggested to be high if the solubility parameter difference between the solute and solvent is low [29,30]. Comparatively higher solubility was observed for TBF-free base in TC, PGML, IPM, OL, and IPA than PG and PEG 200 (*p* < 0.05), and their solubility parameters were close to the solubility parameter value for TBF, 9.87 (cal/cm^3^)^1/2^. The lower solubility of TBF-free base is evident for PEG 200 and PG, consistent with the solubility parameter values of PG, and PEG 200 (14.07 and 12.06 (cal/cm^3^)^1/2^) being more distant from TBF. However, TBF-free base showed higher solubility in EtOH (0.55 ± 0.05 g/mL) than PEG 200 (*p* < 0.05) despite the similarity in solubility parameters for PEG 200 and EtOH (12.27 (cal/cm^3^)^1/2^).

Stability studies of 1% (*w*/*w*) TBF-free base were conducted at 32 ± 1 °C up to 120 h (5 d) for all solvents, and no degradation issues were evident (Figure S4).

### 3.4. Dynamic Vapour Sorption Studies

**Figure 5:**
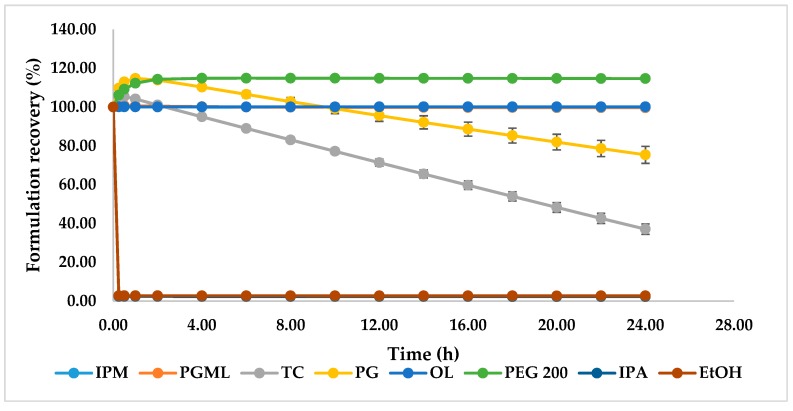
Percentage weight change for 1% (*w*/*w*) TBF-free base formulations in IPM, PGML, TC, PG, OL, PEG 200, EtOH, and IPA at 32 ± 1 °C and 50 ± 1% RH (*n* = 3, mean ± SD).

The DVS results of candidate formulations are shown in Figure 5. As expected, the PGML, IPM, and OL systems showed no change in weight during the study as they are non-volatile [19]. However, for TC and PG, considerable evaporation is evident; an initial spike in weight for these formulations reflects the hygroscopic nature of these solvents [31]. The sustained weight increases for PEG 200 also reflects a similar uptake of water over time. The EtOH and IPA formulations showed rapid evaporation after application, and after 15 min the recovery weights were 2.67% and 2.39% respectively.

### 3.5. In Vitro Finite Dose Neat Solvent and Commercial Formulation Efficacy in Porcine Skin

Finite dose in vitro permeation combined with mass balance studies were conducted for TBF-free base 1% (*w*/*w*) solutions for IPM, PGML, OL, PEG 200, TC, PG, IPA, EtOH, and the commercial preparation. The results are presented in Table 1 for each solvent.

The values for skin surface recovery for all the single solvents (Table 1) indicate that most of the applied TBF-free base was recovered from the skin surface. The skin surface recovery values for IPM, PGML, OL, PEG 200, TC, EtOH, IPA, and commercial preparation accounted for more than 80% of the applied dose. Less than 80% of the active was recovered from the skin surface for the PG solution. PG and TC delivered significantly higher amounts of TBF-free base into the skin compared with the commercial formulation (*p* < 0.05). The percentage skin extraction values for the PG and TC formulations were also almost four times higher than observed for the PGML, OL, and PEG 200 formulations (*p* < 0.05), but were not significantly different compared with IPM (*p* > 0.05). The EtOH and IPA solutions did not deliver a higher amount of TBF-free base to the skin compared with the commercial preparation (*p* > 0.05). The values for percentage skin extraction of TBF for IPM, PGML, OL, and PEG 200 were also not significantly different from the commercial preparation (*p* > 0.05).

PG has been investigated as a penetration enhancer in a range of topical formulations [20,32]. According to the DVS results, the remaining weight for the PG formulation was 75.3% after 24 h (Figure 5). Evaporation of PG after application should increase the thermodynamic activity of TBF-free base and promote skin delivery of TBF-free base. A similar effect has been proposed recently for the skin permeation and uptake of climbazole. Under finite dose conditions, PG enhanced the topical delivery of climbazole in porcine skin compared with PEG 200, octyl salicylate, and TC [31]. The solubility studies (Figure 4) confirmed low solubility of TBF in PG (98.57 ± 1.2 mg/mL). The mechanism of action of PG as a skin permeation enhancer has not been fully elucidated. It is hypothesised that PG may change the partitioning and solubility of permeants inside the membrane [19,20]. As a hydrophilic molecule, PG may integrate between the polar heads of the hexagonally packed lipids, subsequently increasing the distance in the lamellar lipid phase and hence, may increase skin delivery [20,31,33,34]. The effect of PG on the permeation of triprolidine base in human skin has been investigated by Kasting et al. and high permeation was found compared with lipophilic vehicles [20,35]. These researchers suggested that this solvent may alter drug solubility in the skin. Trottet et al. correlated the penetration of PG with the permeation of loperamide hydrochloride under finite dose conditions across human skin in vitro [20,32]. Investigation of the in vivo depth profile of the vehicles and permeants using Confocal Raman spectroscopy also confirmed the penetration dependency of trans-retinol and niacinamide on the penetration of PG [36,37]. The penetration of anthramycin also mirrored the penetration of PG under finite dose in vitro human skin, investigated by Haque et al. [19].

As for PG, TC also delivered a significantly higher amount of TBF-free base to the skin compared with the commercial preparation (*p* < 0.05). The solubility parameter of TC (10.62 (cal/cm^3^)^1/2^) is similar to the proposed value for the skin (10 (cal/cm^3^)^1/2^) [38], and to TBF. It may be hypothesised that this results in high skin interaction of TC with skin. TBF-free base is very soluble in TC (1.19 ± 0.10 g/mL). Thus, high skin uptake of TC facilitates solubilisation of TBF-free base in the skin. From the DVS experiment, 37% of the applied dose of the TC formulation was recovered after 24 h (Figure 5). Again, as for PG, the solvent evaporation should increase the thermodynamic activity of TBF in the formulation and contribute to enhanced skin absorption of TBF-free base. High skin uptake of TC was previously correlated with high uptake of methyl paraben following in vitro studies in human skin [39]. The ability of TC to enhance skin permeation has also been reported for several other actives [19,40,41]. Gannu et al. demonstrated increased flux of carvedilol from a saturated solution in neat TC through porcine skin under infinite dose conditions [41]. These authors also proposed that the solubilisation effect of TC increased the concentration gradient of the drug in the solution, which subsequently favours the partitioning of drug in the stratum corneum. Chadha et al. also found increased flux of genistein across dermatomed human skin from an infinite dose (200 mg) of a gel containing TC compared with the control [40]. The authors suggested that the enhanced flux reflected the ability of TC to facilitate solubilisation of the active in the stratum corneum. Most recently, Haque et al. reported the permeation enhancement of anthramycin from a saturated solution in TC across human skin, under finite dose conditions [19]. As for PG, the authors confirmed that permeation of anthramycin is highly correlated with the penetration of TC.

The percentage of skin retention of applied TBF-free base was similar for EtOH and IPA (*p* > 0.05). EtOH and IPA are short-chain alcohols and are used in a range of topical and transdermal products [20]. It has been reported previously that EtOH and IPA enhanced the permeation and skin retention of various drugs across skin [42,43]. In the present work, EtOH and IPA did not outperform the commercial gel preparation. The DVS studies confirm that only 2.4% and 2.7% of the applied doses of the IPA and EtOH formulations, respectively, were recovered after 15 min (Figure 5). Evaporation of these solvents following application to the skin will limit their contribution to TBF skin delivery. Lower terbutaline flux across split-thickness human skin from neat IPA compared to 60–80% IPA in water has been reported by Liu and Bergstorm [44]. The authors suggested that this reflected the greater dehydration effects of pure IPA compared with the IPA/water systems. The permeation of EtOH across porcine skin has been investigated under unoccluded conditions by Pendlington et al. and the overall recovery of EtOH was 2.2% [45]. The authors suggested surface evaporation contributed to the low flux of EtOH. Oliveira et al. reported low permeation and skin retention of methyl paraben from a saturated finite dose application in EtOH in human skin in vitro. Of the applied dose, ∼94% was recovered from the surface, ∼1% from inside the skin, and 1.8% of the active permeated [46]. The authors also observed the crystallisation of methyl paraben because of rapid evaporation of EtOH, which would restrict the delivery of the active into the skin. Watkinson et al. studied the effect of EtOH on the permeation of ibuprofen across human epidermis and found lower flux for neat EtOH compared to 50% and 75% ethanolic solutions of the active in water [47].

The solubility of TBF-free base in PEG 200 is very low (65.67 ± 1.05 mg/mL). However, the delivery of TBF-free base to the skin is comparable to that observed for the commercial preparation (*p* > 0.05). The solubility parameter of PEG 200 is 12.06 (cal/cm^3^)^1/2^, whereas the reported solubility parameter for the skin is 10 (cal/cm^3^)^1/2^ [19,38]. It is possible that the results reflect low partitioning of PEG 200 into the skin. Oliveira et al. investigated the uptake of methyl paraben by human skin from solutions of the active in IPM, TC, dimethyl isosorbide (DMI), and PEG 200 [39]. The authors reported the lowest skin extraction of methyl paraben from PEG 200 compared with other vehicles. In addition, the DVS studies confirmed that PEG 200 is non-volatile. After application, PEG 200 is expected to remain on the skin surface. Low skin delivery of hexamidine diisethionate and hexamidine hydrochloride salt have been reported previously following application of infinite doses of these actives in PEG 200 in porcine skin by Parisi et al. [48]. Paz-Alvarez et al. also reported lower skin delivery of climbazole from a finite dose of the active in PEG 200 across pig skin compared with TC, PG, and octyl salicylate [31].

IPM, PGML, and OL are widely used penetration enhancers [20]. However, they did not deliver significantly higher amounts of TBF-free base to the skin compared with the commercial preparation (*p* > 0.05). DVS results confirmed that they are non-volatile (Figure 5), and TBF is freely soluble in these solvents (Figure 4). Thus, the active is expected to have comparatively low thermodynamic activity in IPM, PGML, and OL, impacting the delivery of TBF into the stratum corneum.

TBF-free base has favourable physicochemical properties for topical penetration. However, with the exception of IPA, no permeation of the molecule was observed up to 24 h for all the neat solvents. TBF has been reported to bind to keratin [16,49,50,51,52], and therefore may not penetrate past the stratum corneum. This may explain the absence of permeation in the receptor compartment for most of the formulations studied. Keratin accounts for over 80% of the weight of the proteins in the SC [53]. Uchida and Yamaguchi reported that TBF had a high affinity to keratin, and the extent of binding was proportional to the keratin concentration [52]. Tatsumi et al. also investigated the affinity of TBF to keratin and found extensive binding (96%) to keratin [49]. Pharmacokinetic studies in human volunteers over a period of eight days reported minimal systemic exposure to TBF, further confirming the low skin penetration of the drug [54]. The absence of TBF-free base in the receptor chamber following topical application in porcine skin confirms the systemic safety of the drug and suggests that the stratum corneum acts as both a barrier and a reservoir for TBF [16].

## 4. Conclusions and Future Work

Currently, TBF is available in formulations either as the hydrochloride salt or the free base. There is limited information in the literature about the physicochemical properties of the free base, and this was addressed in the first stage of this work. The characterisation of the physicochemical properties of the TBF-free base provided all the necessary information for the rational design of simple formulations with the potential to deliver the active to the skin. However, in conventional Franz cell experiments, for most of the solvents examined, the molecule was not detected in the receptor fluid after 24 h. As noted in the literature, this may reflect an interaction of TBF with the keratin of stratum corneum. For antifungals, skin retention is more desirable than permeation, considering the intended use of active, and the active should act at the site of application. The mass balance studies confirmed that most of the applied doses were recovered from the skin surface for all the tested formulations. The results also confirm that PG and TC were the best solvents for the delivery of TBF-free base to the skin compared with the commercial formulation. Future work will explore in vitro permeation and mass balance studies of binary, ternary, and more complex formulations.

## Figures and Tables

**Table 1:**
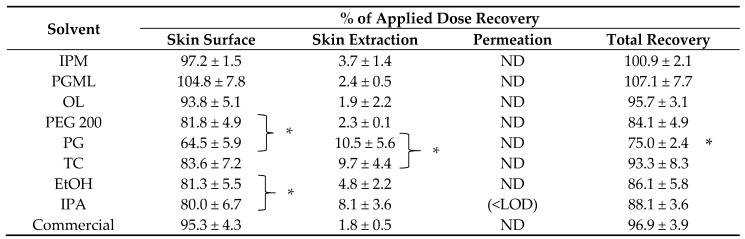
Percentage of TBF-free base recovered from the skin surface, following skin extraction and percentage permeation for finite doses of 1% (*w*/*w*) TBF-free base formulations and commercial control in porcine skin (4 ≤ *n* ≤ 5, mean ± SD).

IPM = Isopropyl myristate; PGML = Propylene glycol monolaurate; OL = Oleic acid; PEG 200 = Polyethylene glycol 200; PG = Propylene glycol; TC= Transcutol^®^; EtOH = Ethanol; IPA = Isopropyl alcohol; ND = Not detected; LOD = Limit of detection; * *p* < 0.05.

## Supplementary Materials

The following are available online at https://www.mdpi.com/1999-4923/11/10/548/s1, Figure S1: ^13^**C** NMR spectrum of TBF-free base, Figure S2: ^13^**C** NMR spectrum of TBF salt, Figure S3: TGA (**a**) and DSC (**b**) analysis of TBF salt, Figure S4: Recovery (%) of TBF-free base in a series of solvent systems after 24, 48, 72, 96, and 120 h at 32 ± 1 °C (*n* = 3; mean ± SD).
