# Supplementary material for: Preparation, Characterisation, and Topical Delivery of Terbinafine
Source: Pharmaceutics. 2019 Oct 22;11(10):548. doi: 10.3390/pharmaceutics11100548 (PMC6835747; doi:10.3390/pharmaceutics11100548)
Supplement: Supplementary file 1 [file pharmaceutics-11-00548-s001.pdf]

pharmaceutics

Supplementary Materials: Preparation, Characterisation, and Topical Delivery of Terbinafine

A.S.M. Monjur Al Hossain, Bruno C. Sil, Fotis Iliopoulos, Rebecca Lever, Jonathan Hadgraft and Majella E. Lane

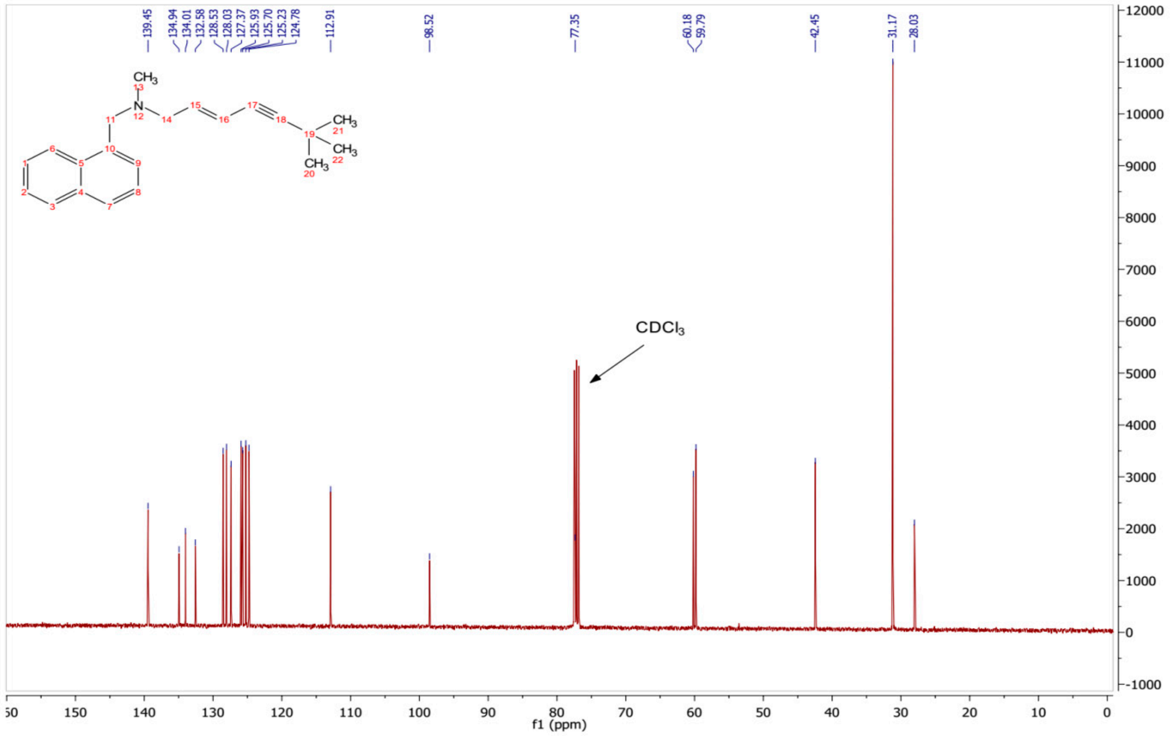

Figure S1. ^{13}C NMR spectrum of TBF base.

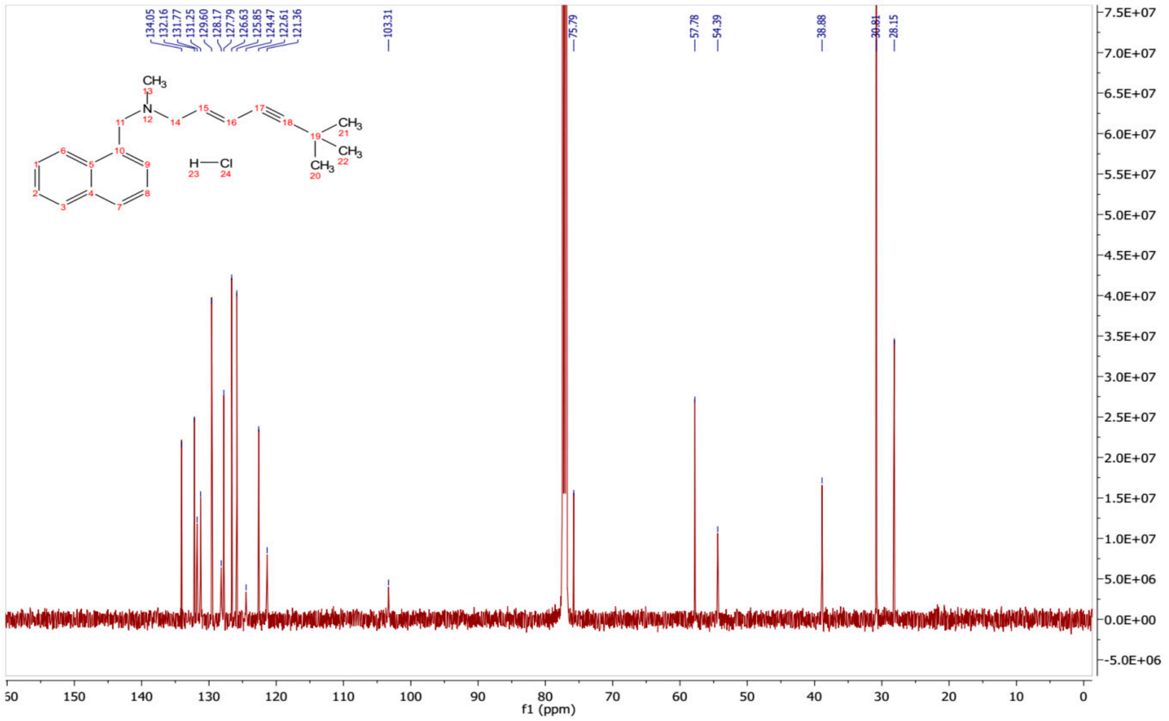

Figure S2. ^{13}C NMR spectrum of TBF salt.

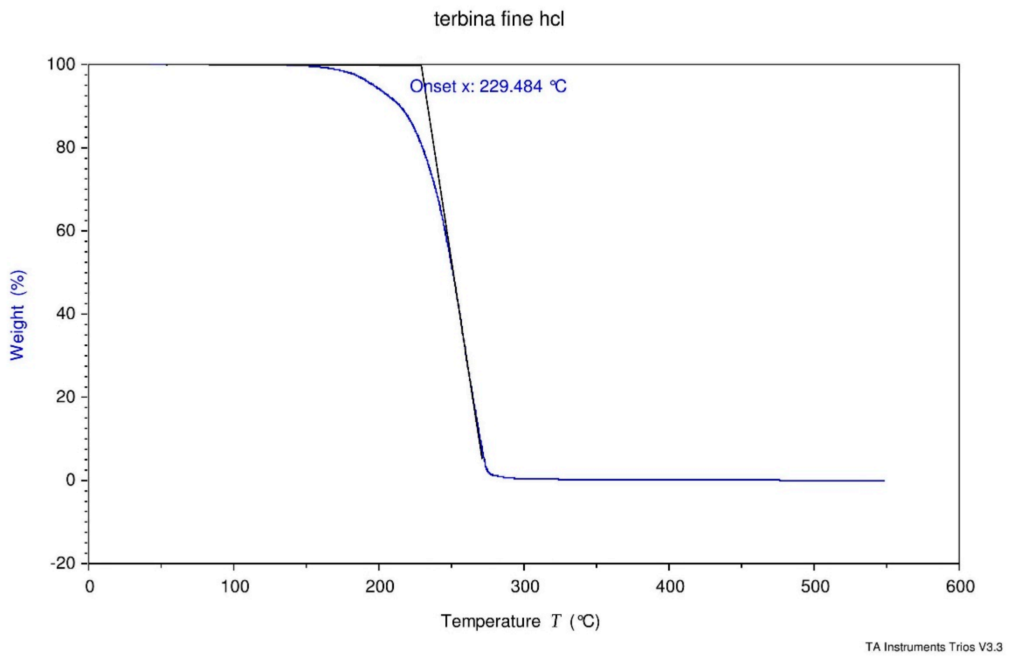

(a)

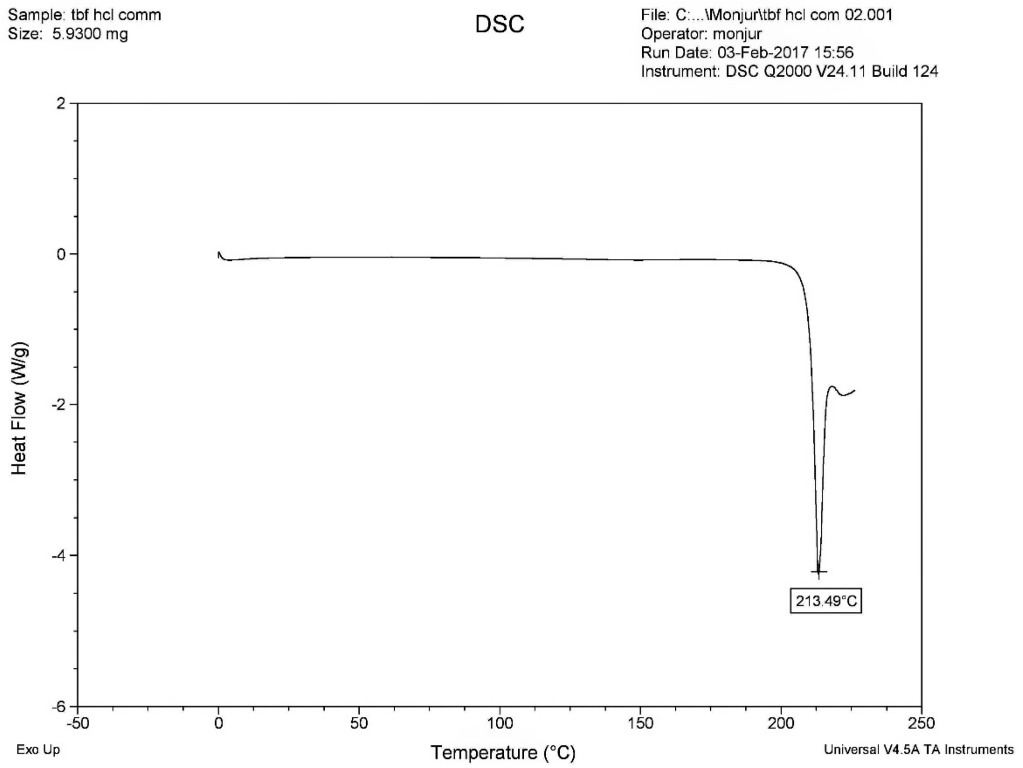

(b)

Figure S3. TGA (**a**) and DSC (**b**) analysis of TBF salt.

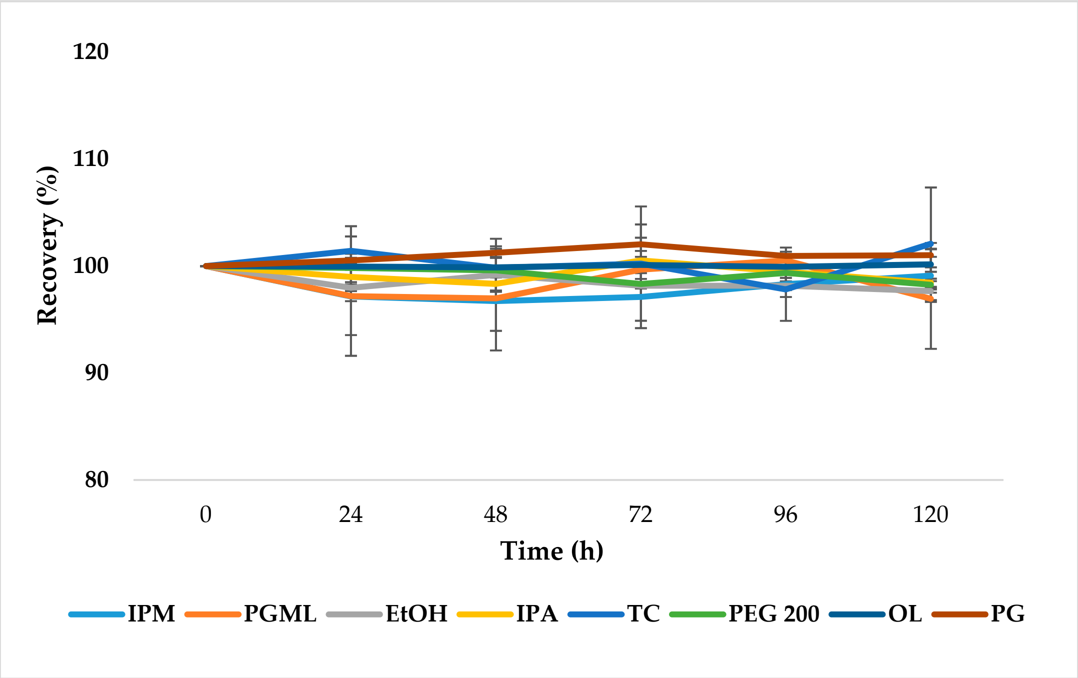

Figure S4. Recovery (%) of TBF-free base in a series of solvent systems after 24, 48, 72, 96 and 120 h at 32 ± 1°C (*n* = 3; mean ± SD).

© 2019 by the authors. Submitted for possible open access publication under the terms and conditions of the Creative Commons Attribution (CC BY) license (http://creativecommons.org/licenses/by/4.0/).
